# Fatal systemic disorder caused by biallelic variants in *FARSA*

**DOI:** 10.1186/s13023-022-02457-9

**Published:** 2022-08-02

**Authors:** Soo Yeon Kim, Saebom Ko, Hyunook Kang, Man Jin Kim, Jangsup Moon, Byung Chan Lim, Ki Joong Kim, Murim Choi, Hee-Jung Choi, Jong-Hee Chae

**Affiliations:** 1 Department of Genomic Medicine, Rare Disease Center, Seoul National University Children’s Hospital, Seoul National University College of Medicine, 101 Daehakro Jongno-gu, Seoul, 110-744 Korea; 2 School of Biological Sciences, Seoul National University, Seoul, Korea; 3 Department Pediatrics, Pediatric Neuroscience Center, Seoul National University, Seoul, Korea; 4 Department of Biomedical Sciences, Seoul National University College of Medicine, Seoul, Korea

**Keywords:** FARSA, Phenylalanyl-tRNA synthetase, Aminoacyl-tRNA synthetase

## Abstract

**Background:**

Aminoacyl tRNA transferases play an essential role in protein biosynthesis, and variants of these enzymes result in various human diseases. *FARSA,* which encodes the α subunit of cytosolic phenylalanyl-tRNA synthetase, was recently reported as a suspected causal gene for multiorgan disorder. This study aimed to validate the pathogenicity of variants in the *FARSA* gene.

**Results:**

Exome sequencing revealed novel compound heterozygous variants in *FARSA*, P347L and R475Q, from a patient who initially presented neonatal-onset failure to thrive, liver dysfunction, and frequent respiratory infections. His developmental milestones were nearly arrested, and the patient died at 28 months of age as a result of progressive hepatic and respiratory failure. The P347L variant was predicted to disrupt heterodimer interaction and failed to form a functional heterotetramer by structural and biochemical analyses. R475 is located at a highly conserved site and is reported to be involved in phenylalanine activation and transfer to tRNA. The R475Q mutant FARSA were co-purified with FARSB, but the mutant enzyme showed an approximately 36% reduction in activity in our assay relative to the wild-type protein. Additional functional analyses on variants from previous reports (N410K, F256L, R404C, E418D, and F277V) were conducted. The R404C variant from a patient waiting for organ transplantation also failed to form tetramers but the E418D, N410K, F256L, and F277V variants did not affect tetramer formation. In the functional assay, the N410K located at the phenylalanine-binding site exhibited no catalytic activity, whereas other variants (E418D, F256L and F277V) exhibited lower ATPase activity than wild-type FARSA at low phenylalanine concentrations.

**Conclusions:**

Our data demonstrated the pathogenicity of biallelic variants in *FARSA* and suggested the implication of hypomorphic variants in severe phenotypes.

**Supplementary Information:**

The online version contains supplementary material available at 10.1186/s13023-022-02457-9.

## Introduction

Aminoacyl-tRNA synthetases (ARSs) play an essential role in protein biosynthesis by linking tRNAs to their cognate amino acids. These enzymes are encoded by 37 nuclear genes, which have all been reported as causes of human Mendelian disorders [1–7]. The FARS1, a cytosolic phenylalanyl-tRNA synthetase, is a heterotetramer with two α and two β subunits encoded by *FARSA* and *FARSB*, respectively [8]. Biallelic variants in *FARSB* were demonstrated to be causative of Rajab syndrome (MIM#613658), which presents with hypotonia, psychomotor retardation, liver dysfunction and involvement of the lung and skeletal systems [4, 9]. A similar phenotype in a boy with biallelic variants in *FARSA* was first described in 2019 [7]. To the best of our knowledge, 21 patients with FARS1-related disorders have been reported, with limited associated functional data, suggesting a variable disease pathomechanism [4, 7, 9–12]. Although the patients shared characteristic phenotypes, they exhibited varying disease severity [9, 10]. Further research on the detailed molecular pathomechanism combined with clinical observations is necessary to understand this disease.

Here, we describe a severe case with biallelic variants in *FARSA* and provide in vitro functional evidence.

## Result

### Case summary and literature review

**Fig. 1:**
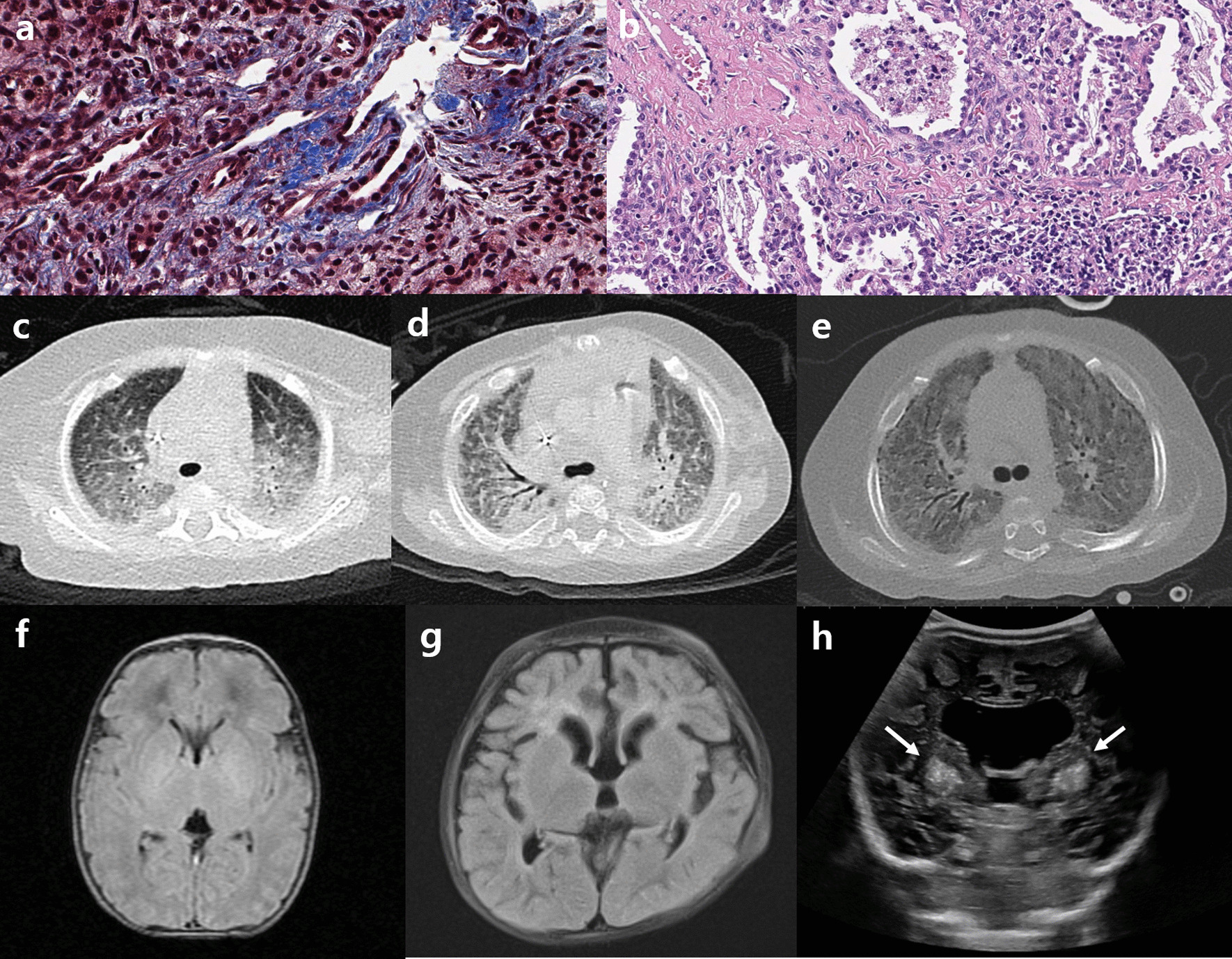
Pathologic and imaging findings of the patient. **a** Liver biopsy indicated periportal fibrosis and diffuse infiltration of inflammatory cells. **b** Lung tissues showed interstitial fibrosis with alveolar thickening. **c**–**e** Sequential chest CT revealed progressive diffuse ground-grass opacity. **f**–**g** Brain imaging indicated progressive brain atrophy, **h** bilateral calcifications at the basal ganglia (arrows)

The index patient was born at a gestational age of 34^+5^ weeks via emergent cesarean section because of maternal preeclampsia. His birth weight was 1.54 kg, and he was admitted to a neonatal intensive care unit for 1 month. He was readmitted for poor weight gain and unexplained pancytopenia at the age of 5 months. He was hypotonic and had delayed motor milestones. Laboratory tests indicated elevated liver enzymes and tubulopathy. By the age of 14 months, liver dysfunction had progressed to hepatic failure with cirrhotic changes, as confirmed by liver biopsy (Fig. 1a, b). Respiratory infections which required mechanical respiratory support and combined acute azotemia occurred repeatedly from the beginning of hospitalization, and he became dependent on 24-h oxygen at the age of 12 months. Computed tomography of the chest revealed extensive bilateral ground-glass opacity in the lungs. Lung biopsy performed at 19 months of age indicated diffuse interstitial thickening and fibrosis with focal cystic change and mild lymphocytic infiltration (Fig. 1c–e). Magnetic resonance imaging of the brain indicated brain atrophy at 11 months of age (Fig. 1f, g), and brain sonography performed at 20 months of age revealed increased echogenicity in both the basal ganglia, the pontine tegmentum and the dentate nucleus, suggesting calcification (Fig. 1h). He was hospitalized for over 2 years and was on a waiting list for liver and lung transplantations. Exome sequencing (ES) identified novel compound heterozygous variants, c.1040C > T (P347L) and c.1424G > A (R475Q), in *FARSA* (Additional file 1). After molecular diagnosis and comprehensive counselling, the parents declined to continue life-sustaining treatment, and the patient died at the age of 28 months. We identified 4 additional patients with *FARSA* variants through a literature review (Table 1) [7, 9]. All patients exhibited the common but various degrees of clinical symptoms of failure to thrive, hepatic dysfunction and progressive interstitial lung disease, although the age of onset and progression varied among them. The phenotypic comparison based on different literatures was challenging because authors might describe patients from different viewpoints in concise text. We compare the phenotypes based on some keys such as major organ involvements and their onset age, developmental milestones, final basic performance, and necessity of organ transplantations. The index patient had been bedridden throughout his life, and his hepatic and pulmonary dysfunction required organ transplantations despite of intensive care for 2 years. Therefore we decided the index case showed the most severe clinical course compared with other cases, except the Patient 4, who died of respiratory failure at 1.3 years old. To understand the molecular basis of variants, we performed a structural analysis and biochemical characterization of each variant in *FARSA*.

**Table 1:** Clinical features of patients with FARSA-related disorder

	Patient 1 (Present study)	Patient 2 (Krenke et al.)	Patient 3 (Schuch et al.)	Patient 4 (Schuch et al.)	Patient 5 (Schuch et al.)
Sex	Male	Male	Female	Female	Male
Age at last examination	Died at 28 months	15 years	17 years	Died at 1.3 years	Died at 12.9 years
*FARSA* genotype					
Variant 1	c.1424G > A (R475Q)	c.776 T > C (F256L)	c.1210C > T (R404C)	c.883C > T (R295W)	c.829 T > G (F277V)
Variant 2	c.1040C > T (P347L)	c.1230C > A (N410K)	c.1254G > C (E418D)	c.883C > T (R295W)	c.829 T > G (F277V)
Central nervous system					
Neonatal hypotonia	+	+	+	+	–
Motor delay	+	+	+	+	–
Speech delay	+	–	+	+	–
Brain imaging	Diffuse atrophy and calcifications at both deep grey matter	Subcortical calcifications Periventricular cysts	Multiple subcortical white matter lesions Elongated brain arteries	Normal by 1 month	Arachnoid cyst
Nutrition and growth					
Feeding intolerance	+	+	–	+	–
Poor weight gain	+	+	+	+	+
Vomiting and diarrhoea	+	+	–	+	–
Lung involvement					
Recurrent respiratory infection	+	+	+	+	+
Interstitial lung disease	+	+	+	+	+
Cholesterol pneumonitis	–	+	+	+	+
Cystic lung disease	–	–	–	–	+
Others					
Renal involvement					
Proteinuria	+	–	–	–	–
Tubulopathy	+	–	–	+	–
Vesicourethral reflux	–	+	–	–	–
Others	Increased renal echogenicity	–		–	Nephrolithiasis
Hepatic involvement					
Abnormal liver function	+	+	+	+	+
Hepatosplenomegaly	+	+	+	+	–
Liver steatosis	+	+	+	+	–
Liver fibrosis	+	–	–		
Hormonal insufficiency	Subclinical hypothyroidism Adrenal insufficiency	Hypopituitarism	Growth hormone resistance	–	Growth hormone deficiency
Dysmorphism/malformation	–	Deep-set eyes, elfin-like face	–	–	–
Skeletal involvement	–	Scoliosis, arachnodactyly, pectus carinatum	Hyperflexible joints Arachnoid fingers	–	Pectus carinatum, hyperflexible joint
Others	Pancytopenia	Left inguinal hernia Microcytic anaemia	–	Intermittent nystagmus	Sensorineural hearing loss

### Structural analysis and biochemical characterization of FARSA variants

**Fig. 2:**
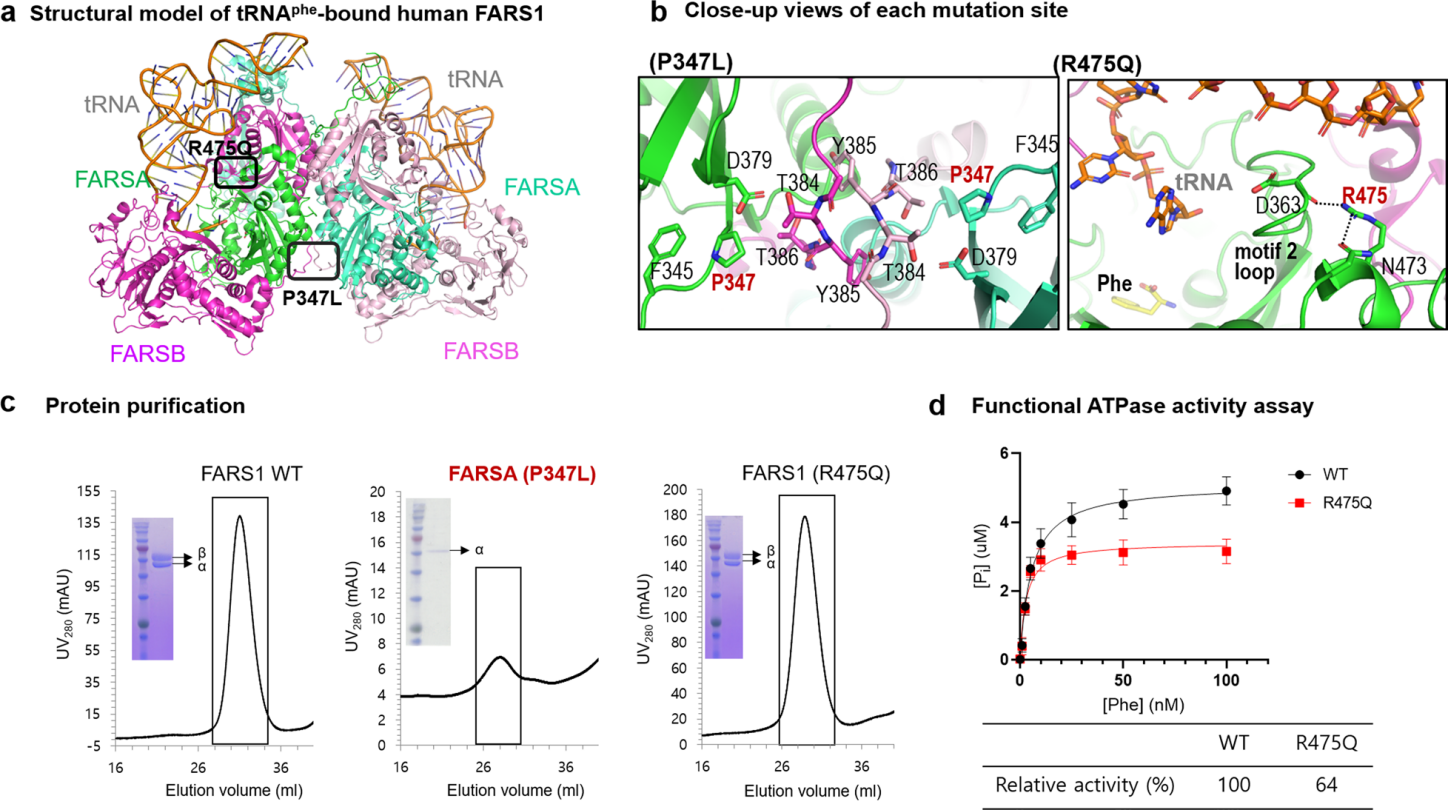
The effects of P347L and R475Q variants of *FARSA* on structural integrity and function. **a** The structural model of tRNA^Phe^-bound human FARS1 (PDB ID 3L4G) is represented as a ribbon diagram. The location of each variant is marked with a black box in this model. **b** Close-up views of each variant site are presented. Residues interacting with P347 (left) and R475 (right) are shown as stick figures, and dotted lines indicate polar interactions. **c** Purification profiles and SDS–PAGE gels showing purified heteromers (WT and R475Q) and FARSA P347L mutant that failed to form a heteromer with FARSB are presented. **d** In vitro ATPase activity assays of wild-type (WT) and R475Q FARSA variants revealed reduced activity of R475Q compared with wild-type FARSA. Three independent experiments, each in triplicate, were performed for each dataset

The location of each variant was annotated in the modelled structure (Fig. 2a and Additional file 2). P347 is located at the heterodimer interface, where P347 and D379 of FARSA interact with T384 of FARSB (Fig. 2b). Substitution of Pro with Leu at position 347 disrupts the heterodimer interface, which results in failure to form a stable heterotetramer. Consistent with this structural analysis, we found that the P347L could not form a heterotetramer with FARSB, whereas wild-type and R475Q mutant FARSA were co-purified with FARSB, to form heterotetramers (Fig. 2c).

R475 is in a highly conserved site. It forms polar interactions with N473 and the carbonyl group of D363, which is located at the ‘motif 2 loop’ (Fig. 2b). Previously, the motif 2 loop was reported to be involved in Phe activation and transfer to tRNA^Phe^. The substitution with glutamine may weaken these polar interactions, especially with the carbonyl of D363. Our in vitro functional analysis showed that the R475Q variant presented a ~ 36% reduction of activity relative to the wild-type protein (Fig. 2d).

**Fig. 3:**
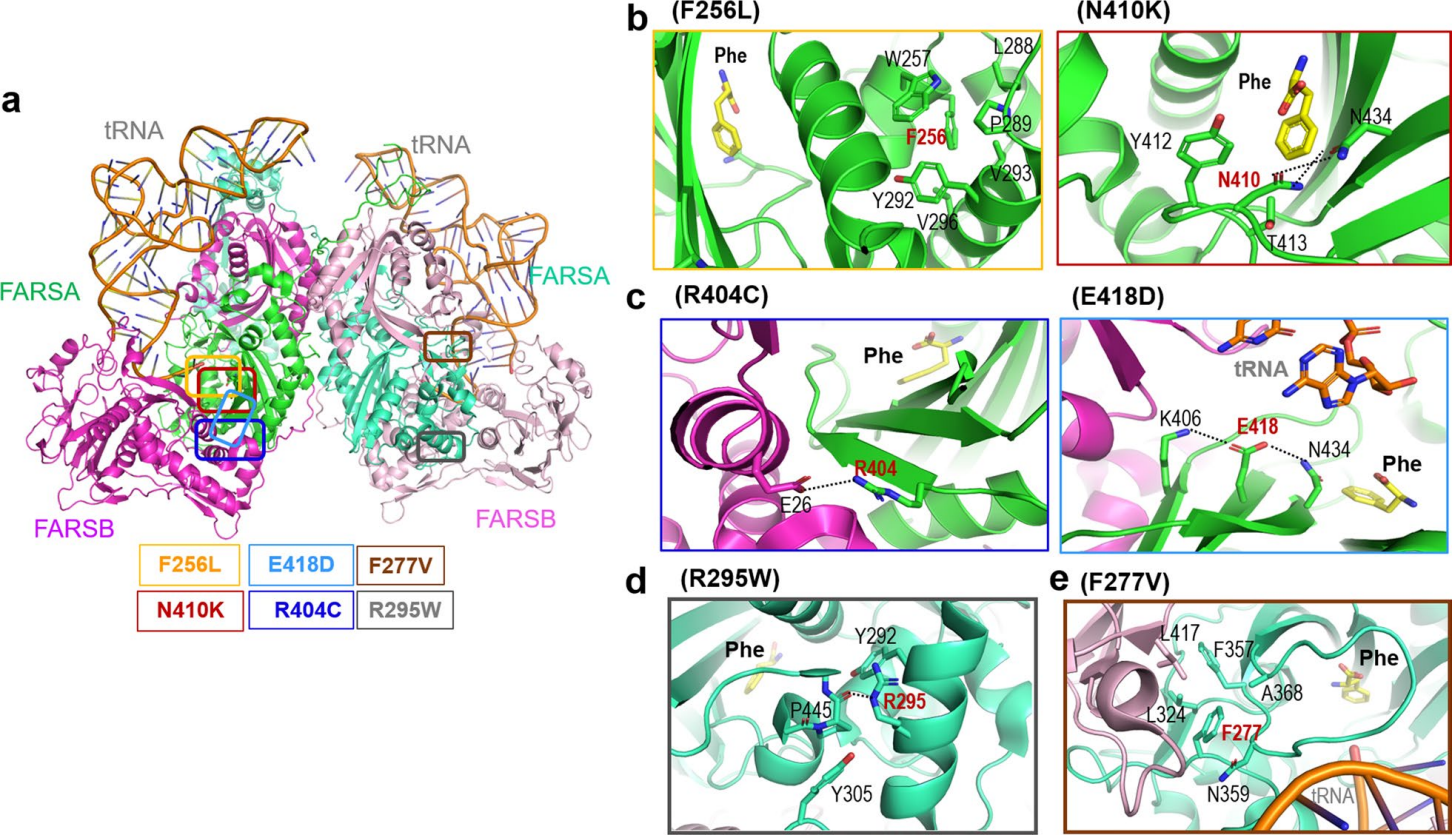
**a** The previously reported variant positions in FARSA are indicated by colored boxes in the modeled structure. **b**–**e** Residues interacting with each mutant residue are shown as stick figures; **b** F256L and N410K of Patient 2, **c** R404C and E418D of Patient 3, **d** R295W of Patient 4, and **e** F277V of Patient 5. Dotted lines indicate polar interactions

**Fig. 4:**
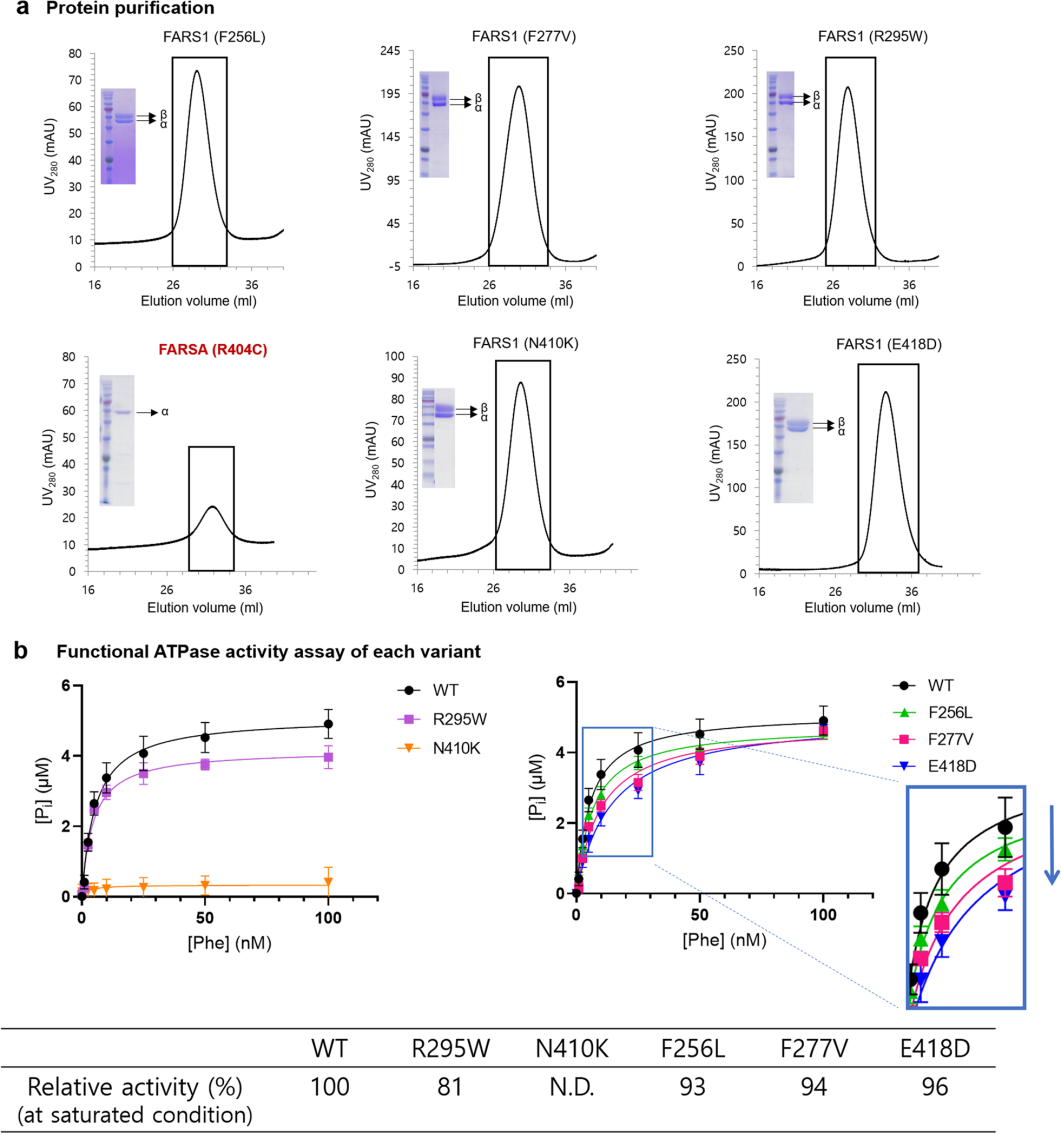
**a** Purification profiles from heparin chromatography and SDS–PAGE gels showing purified protein bands of each variant. Most of the variants displayed similar profiles as wild-type FARSA, while the R404C mutant FARSA was not copurified with FARSB, similar to the P347L mutant. **b** In vitro ATPase activity of each purified variant was measured with a malachite green phosphate assay. Each dataset was collected in triplicate at eight different concentrations of l-*Phe*. Three independent experiments were performed for each dataset. The activity of each mutant was expressed relative to the wild-type activity (100%). N.D. means no activity detected. The F256L, F277V, and E418D mutants showed wild-type-like activity (more than 90% of wild-type activity) at saturated *Phe* concentrations, but their activities were significantly reduced compared with wild-type activity at low *Phe* concentrations, as shown in the enlarged graph on the right

We performed additional functional analyses of other *FARSA* variants from previous studies (Table 1, Figs. 3, 4, and Additional file 3). Structural analyses on variants showed similar conclusions with previous report [9]. F256L and N410K had no effect on the formation of a heterotetramer (Figs. 3b, 4). N410 is located at the Phe substrate-binding site, and its substitution with Lys would disrupt the active-site structure, thus interfering with Phe binding (Fig. 3b). Accordingly, a functional assay revealed that N410K exhibited no catalytic activity (Fig. 4). F256L, in contrast, was predicted to have no significant effect on the FARSA structure or Phe binding activity, since the Leu residue could still participate in hydrophobic network formation with nearby hydrophobic residues (Fig. 3b). However, Leu cannot form π–π interactions with W257 and Y292 as Phe does, which may affect structural stability. In our functional assay, we observed that F256L exhibited lower activity than wild-type FARSA at low Phe concentrations (Fig. 4). In case of the Patient 3, The structural analysis suggests that the R404C variant would interfere with heteromer formation, as R404 is located at the binding interface between the α and β subunits, forming a salt bridge with E46 of FARSB (Fig. 3c). A previous report similarly noted the effect of the R404C variant on heteromer interactions [9]. Protein purification of FARS1 containing the R404C variant demonstrated that R404C caused failure to form a heterotetramer with FARSB, similar to P347L (Fig. 4).

The Patient 4 and 5 had homozygous variants. Our in vitro study showed that R295W formed a stable heterotetramer similar to wild-type FARSA but with 20% lower activity than wild-type (Figs. 3, 4). Another homozygous F277V variant behaved similarly to E418D and F256L, exhibiting reduced activity only at low Phe concentrations (Fig. 4).

## Discussion

Patients with *FARSA* or *FARSAB* variants presented similar multi-organ involvements with different clinical course. All reported patients have lung involvement, but they showed different onset and severity [9, 12]. Patients also often has liver dysfunction, growth failure, developmental delay, and skeletal problems in different ways [9]. Some patients showed progressive vascular leukoencephalopathy with recurrent haemorrhages or strokes [12]. Here, we described one of the most severe case with novel *FARSA* variants and provided biochemical and functional data. A similar devastating clinical course was reported in a patient with biallelic variants, c.767C > T and c.1486delCinsAA, in *FARSB* [4]. This patient presented early-onset and rapidly progressing interstitial lung disease and liver cirrhosis. He also exhibited pancytopenia, diffuse brain atrophy and pulmonary hypertension, and he passed away at 32 months of age. The c.1486delCinsAA variant is a null allele, and the c.767C > T variant was also predicted to cause structural instability. Western blot analysis using fibroblasts of the patient indicated a dramatic reduction in protein expression [4]. No clinical observations of homozygous or compound heterozygous null variants in *FARSA* or *FARSB* have been reported to date, and missense variants account for the majority of the reported variants, even in other ARS-related disorders. Some animal studies on ARSs suggest high embryonic lethality after complete knockout of the genes of interest [13, 14]. In the present study, we showed the P347L variant is nonfunctional, as it is unable to form stable heterotetramer with FARSB. Furthermore, we demonstrate that FARS1 with the R475Q variant in FARSA has reduced activity (64% of wild-type activity). Based on these data, we suggest that our severely hypomorphic variant caused severe disease manifestations.

The variants, N410K and F256L, did not affect heterotetramer formation, and the patient had normal intelligence and mild clinical course as he survived until 15 years of age without profound organ failure or cognitive impairment. Patient 3, with R404C and E418D, showed relatively late-onset hepatic steatosis and interstitial lung disease. The patient survived until 17 years old but her pulmonary function significantly deteriorated, which made the patient a candidate for lung transplantation. We demonstrated that the R404C caused failure to form a heterotetramer with FARSB. In the index case and Patient 3, FARSB was coexpressed with each mutant FARSA but not copurified (Additional file 3). We suspect that the phenotypic differences (slower course than the index case) might originate from the other variant, E418D, which showed near-normal activity under Phe saturation conditions (Fig. 4). Of note, at low Phe concentrations, E418D exhibited reduced activity (Fig. 4).

Based on our in vitro studies, we could explain to some extent phenotypic severity as the effect of variants on structural integrity and function. However, in some cases, in vitro functional assays alone could not account for variant-induced disease severity in vivo. Patient 3, 4, and 5 from previous report (Table 1) showed normal protein expression in Western blots using fibroblasts, which suggested pathomechanisms other than direct impairment of FARS1 activity [9]. Xu et al. suggested a nontranslational mechanism based on normal aminoacylation activity in patients with c.848 + 1A and R305Q variants in *FARSB* [10]. Recent study indicated abnormal inflammatory profiles in patients with *FARSA* variants [12]. There have also been other experimental reports on some ARSs that were identified to have diverse functions, including cell signaling [15–17]. Some organ-specific features and grouped phenotypes depending on the type of ARS also suggest a further functional effect on homeostasis [1, 14, 18–20]. Our functional analyses for homozygous variants could not provide clear evidences for phenotypic severity. Additional studies are required to understand the detailed pathomechanism underlying FARS1-related disorders.

## Conclusion

We described a case with biallelic variants in *FARSA* and elucidated their functional effects on disease severity. Overall, there might be additional pathomechanisms underlying FARS1-related disorders, but our data demonstrated the structural effect and dysfunction of each variant, supporting their clinical association with disease severity.

## Methods

The patient was a participant in the Korean Undiagnosed Disease Program and underwent ES. The entire protocol was approved by the Institutional Review Board (IRB) of the Seoul National University Hospital (IRB No. 1904-054-1027). The patient and his legal representatives provided written informed consent. Functional analyses was performed using biochemical and structural analyses and in vitro phenylalanine-binding assays. We also reviewed previous literatures of patients with *FARSA* or *FARSB* variants, to compare the phenotype and evaluate their variants. We searched PubMed using advanced search builder, between database inception and Jun 2022. We used terminologies of FARSA, FARSB, FARS1, phenylalanine-tRNA synthetase, and phenylalanine-tRNA synthetase deficiency as independent searching terminology.

### DNA preparation and exome sequencing

Genomic DNA was extracted from peripheral blood leucocytes using a QIAamp DNA Blood Midi Kit according to the manufacturer’s instructions (Qiagen, Valencia, CA, USA). Capture probes based on SureSelect Human All Exon V5 (Agilent Technologies, Santa Clara, CA, USA) were used for sequencing. The library was paired-end sequenced with a HiSeq 2500 sequencing system (Illumina, San Diego, CA, USA). The sequenced reads were aligned to the Genome Reference Consortium Human Build 37 (patch release 13) using the Burrows–Wheeler Aligner (version 0.7.15). The Picard software (version 2.8.0), SAMtools (version 1.8) and the Genome Analysis Toolkit (GATK, version 4.1.4) were used for further data processing. All variants were called using the GATK HaplotypeCaller in the GVCF mode, and the called variants were annotated using ANNOVAR, SnpEff and InterVar. The pathogenicity of variants was evaluated according to the American College of Medical Genetics standard guidelines [21].

### Protein purification

The cDNAs of *FARSA* and *FARSB* were cloned into a modified pETDuet-1 vector for dual protein expression in *Escherichia coli*. To purify heteromers using Ni–NTA, constructs were designed with a His_6_-tag at the N-terminus of FARSA and no affinity tag at FARSB. Coexpression of FARSA carrying a His_6_-tag at the N-terminus and FARSB in *E. coli* Rosetta (DE3) cells was induced using 0.5 mM isopropyl β-d-1-thiogalactopyranoside (IPTG) (Promega, Madison, WI, USA), and the cells were harvested after further incubation at 25 °C for 12 h. For protein purification, cells were lysed in lysis buffer (20 mM Tris–HCl pH 8.5, 500 mM NaCl, 1 mM phenylmethylsulfonyl fluoride (PMSF) (Roche, Basel, Switzerland) with 20 U of DNase 1 (Roche, Basel, Switzerland)) using Emulsiflex C3 (Avestin, Ottawa, Canada). After the cell lysate was cleared by centrifugation at 14,000 rpm for 15 min, the supernatant was loaded onto a Ni–NTA column (QIAGEN, Germantown, MD, USA). Bound proteins were eluted with elution buffer (20 mM Tris–HCl pH 8.5, 100 mM NaCl, 300 mM imidazole and 0.1 mM TCEP) after column washing with wash buffer A (20 mM Tris–HCl, pH 8.5, 500 mM NaCl, 20 mM imidazole and 0.1 mM tris (2-carboxyethyl) phosphine (TCEP) (Hampton Research, Aliso Viejo, CA, USA)) and wash buffer B (20 mM Tris–HCl, pH 8.5, 150 mM NaCl, 30 mM imidazole and 0.1 mM TCEP). To remove the His_6_-tag, HRV3C protease was treated overnight at 4 °C. Further purification was carried out using a HiTrap Heparin HP column (Cytiva, Marlborough, MA, USA) equilibrated with buffer A (20 mM Tris–HCl pH 8.5, 1 mM EDTA and 1 mM dithiothreitol (DTT) (Thermo Fisher Scientific, Waltham, MA, USA)). Proteins were eluted using a 10–40% linear gradient of NaCl and further purified via size-exclusion chromatography (Cytiva, Marlborough, MA, USA) equilibrated with 20 mM HEPES–NaOH pH 7.5, 150 mM NaCl, 0.1 mM EDTA and 1 mM DTT. The purified proteins were concentrated and stored at − 80 °C until use.

Except for P347L and R404C mutants of FARSA, all other FARSA proteins were copurified as heteromers with FARSB. To confirm that FARSB was well expressed but not copurified with the P347L and R404C variants, the FARSB construct was newly designed to contain a His_6_-tag at the C-terminus, and its expression was tested in *E. coli* Rosetta (DE3) cells. As shown in Supplementary Figure S4, His-tagged FARSB was purified by Ni–NTA but did not form heteromers with FARSA with P347L or R404C variant.

### Functional ATPase activity assay

Aminoacylation of tRNA occurs in two steps mediated by FARS. First, FARS hydrolyses ATP to activate Phe with AMP; second, it transfers activated Phe to tRNA^Phe^. The first step of aminoacylation was monitored using a *malachite green phosphate assay* system (Sigma–Aldrich, St. Louis, MO, USA) [22]. Assays were performed in reaction buffer (20 mM HEPES–NaOH pH 7.5, 150 mM NaCl, 30 mM KCl, 4 mM MgCl_2_ and 1 mM DTT) with 400 μM ATP (Sigma–Aldrich, St. Louis, MO, USA), 1 μM purified FARS, 5 U/ml PPase (Sigma–Aldrich, St. Louis, MO, USA)) and various concentrations (0–100 μM) of l-Phe (Sigma–Aldrich, St. Louis, MO, USA). After 30 min of incubation at 37 °C, reactions were stopped with 5 mM EDTA. Each quenched sample was mixed with malachite green solution in flat-bottom 96-well clear plates (SPL, Seoul, Korea) and incubated for 30 min at room temperature. Absorbance was then measured at a wavelength of 620 nm using a FlexStation3 Multiplate reader (Molecular Devices, San Jose, CA, USA). Each dataset was collected in triplicate at eight different concentrations of l-Phe and subsequently analyzed using GraphPad Prism 8.4.3 (San Diego, CA, USA). Three independent experiments were performed on each dataset.

### Structural analysis of FARS1 variants and modeling of the structure of the human FARS–tRNA^Phe^ complex

Functional FARS is a tetramer, (αβ)_2_, formed by a heterodimer consisting of FARSA (α subunit) and FARSB (β subunit) (Additional file 2) [23]. The crystal structure of human cytosolic FARS1 with a Phe substrate (PDB ID 3L4G) was previously reported, revealing the structural integrity of human FARS1 and the molecular details of Phe substrate binding [8]. However, the structure of tRNA^Phe^-bound human FARS1 has not been reported yet. Thus, the structure of FARS1–tRNA^Phe^ complex was modelled by using the crystal structure of *tt*FARS–tRNA^Phe^ complex (PDB ID 2IY5), as a template (Additional file 2) [24]. The conserved α subunit of the two structures were aligned using the Coot program, then, tRNA^Phe^ was introduced into the human FARS1 structure. This modeled structure was used to analyze the FARSA variants.

## Supplementary Information

**Additional file 1:** Detailed information of variants.

**Additional file 2:** The crystal structure of human cytosolic FARS1 (PDB ID 3L4G) (a) and the modeled structure of the FARS1–tRNAPhe complex, which was derived from the structure of tRNAPhe–bound Thermus thermophilus FARS (PDB ID 2IY5) (b).

**Additional file 3:** SDS–PAGE gels showing loading, flow-through and elution samples from heparin chromatography of the P347L (left) and R404C (right) mutants FARSA coexpressed with FARSB with a His6-tag are presented. Each heparin column loading sample is the elution sample from Ni-NTA. As both FARSA and FARSB contain a His6-tag, both subunits were captured by a Ni-NTA affinity column but were not copurified by a heparin column because they did not form stable heteromers. In contrast, all other FARSA mutants and wild-type FARSA were copurified with FARSB by a heparin column, as shown in Figure 2c and Supplementary Figure S3.

## Data Availability

The data that support the findings of this study are available on request from the corresponding author. The data are not publicly available due to privacy or ethical restrictions.

## Abbreviations

ES: Exome sequencing
IRB: Institutional Review Board
