# Supplementary material for: Fatal systemic disorder caused by biallelic variants in FARSA
Source: Orphanet J Rare Dis. 2022 Aug 2;17:306. doi: 10.1186/s13023-022-02457-9 (PMC9344665; doi:10.1186/s13023-022-02457-9)
Supplement: Supplementary file 1 — Additional file 1: Detailed information of variants. [file 13023_2022_2457_MOESM1_ESM.docx]

**Supplementary Table S1. Detailed information of variants**

|  | **Position** | **cDNA change** | **Amino acid change** | **Read depth of reference allele** | **Read depth of nonreference allele** | **Inheritance** | **CADD score** |
| --- | --- | --- | --- | --- | --- | --- | --- |
| **Variant 1** | **Chr19:13035608** | **c.1040C>T** | **p.Pro347Leu** | **113** | **98** | **From father** | **29.5** |
| **Variant 2** | **Chr19:13033665** | **c.1424G>A** | **p.Arg475Gln** | **63** | **75** | **From mother** | **35** |
