# Supplementary figures and images for: Fatal systemic disorder caused by biallelic variants in FARSA
Source: Orphanet J Rare Dis. 2022 Aug 2;17:306. doi: 10.1186/s13023-022-02457-9 (PMC9344665; doi:10.1186/s13023-022-02457-9)

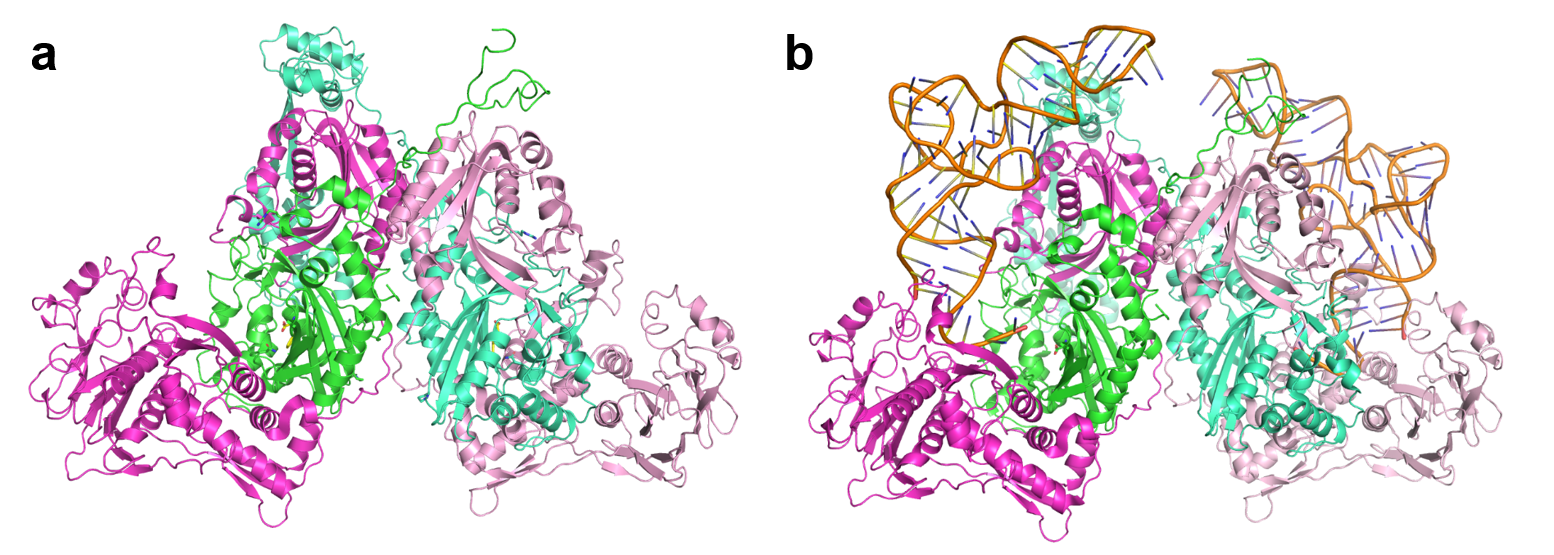

Supplement: Supplementary file 2 — Additional file 2: The crystal structure of human cytosolic FARS1 (PDB ID 3L4G) (a) and the modeled structure of the FARS1–tRNAPhe complex, which was derived from the structure of tRNAPhe–bound Thermus thermophilus FARS (PDB ID 2IY5) (b). [file 13023_2022_2457_MOESM2_ESM.png]

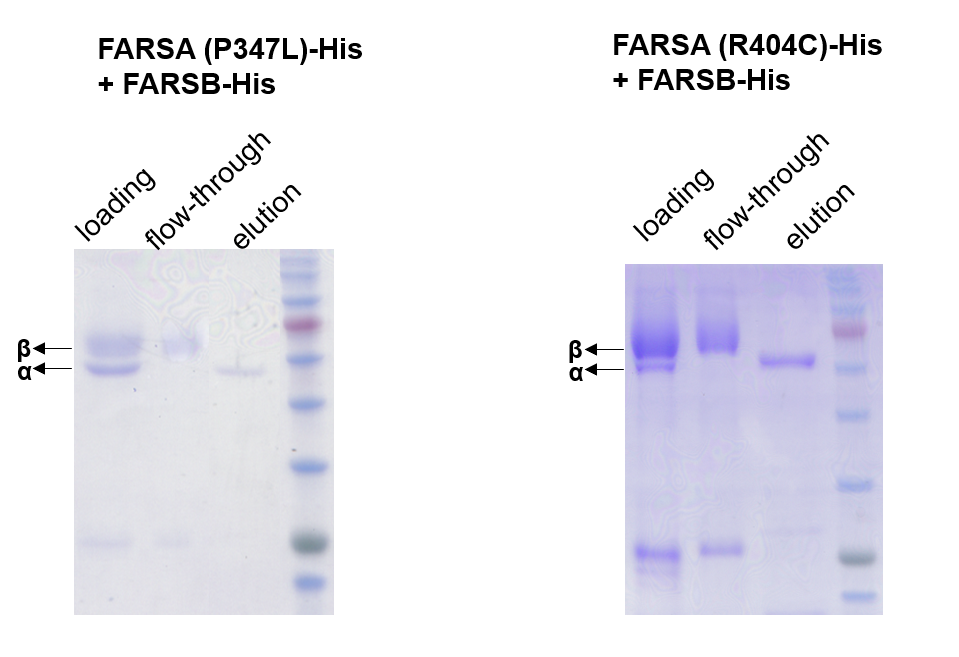

Supplement: Supplementary file 3 — Additional file 3: SDS–PAGE gels showing loading, flow-through and elution samples from heparin chromatography of the P347L (left) and R404C (right) mutants FARSA coexpressed with FARSB with a His6-tag are presented. Each heparin column loading sample is the elution sample from Ni-NTA. As both FARSA and FARSB contain a His6-tag, both subunits were captured by a Ni-NTA affinity column but were not copurified by a heparin column because they did not form stable heteromers. In contrast, all other FARSA mutants and wild-type FARSA were copurified with FARSB by a heparin column, as shown in Figure 2c and Supplementary Figure S3. [file 13023_2022_2457_MOESM3_ESM.png]
